# Neuroimaging-guided diagnosis of possible FTLD-FUS pathology: a case report

**DOI:** 10.1186/s13550-024-01102-9

**Published:** 2024-04-04

**Authors:** Gregory Mathoux, Cecilia Boccalini, Aurelien Lathuliere, Max Scheffler, Giovanni B. Frisoni, Valentina Garibotto

**Affiliations:** 1 Division of Nuclear Medicine and Molecular Imaging, Geneva University Hospitals, Geneva, Switzerland; 2 Laboratory of Neuroimaging and Innovative Molecular Tracers (NIMTlab), Faculty of Medicine, Geneva University Neurocenter, University of Geneva, Geneva, Switzerland; 3 Department of Rehabilitation and Geriatrics, Memory Clinic, Geneva University and University Hospitals, Geneva, Switzerland; 4 Division of Radiology, Geneva University Hospitals, Geneva, Switzerland; 5 Laboratory of Neuroimaging of Aging (LANVIE), University of Geneva, Geneva, Switzerland; 6 CIBM Center for Biomedical Imaging, Geneva, Switzerland; 7 Neuroimaging and Innovative Molecular Traces Lab, Hôpitaux Universitaires de Genève (HUG) & University of Genève, Rue Gabrielle-Perret-Gentil 4, Genève 14, CH-1205 Switzerland

**Keywords:** PET, Neuroimaging, FTLD-FUS pathology, Mild cognitive impairment, Chorea

## Abstract

**Background:**

This case report presents a patient with progressive memory loss and choreiform movements.

**Case presentation:**

Neuropsychological tests indicated multi-domain amnestic mild cognitive impairment (aMCI), and neurological examination revealed asymmetrical involuntary hyperkinetic movements. Imaging studies showed severe left-sided atrophy and hypometabolism in the left frontal and temporoparietal cortex. [^18^F]Flortaucipir PET exhibited moderately increased tracer uptake in hypometabolic areas. The diagnosis initially considered Alzheimer’s disease (AD), frontotemporal degeneration (FTD), and corticobasal degeneration (CBD), cerebral hemiatrophy syndrome, but imaging and cerebrospinal fluid analysis excluded AD and suggested fused-in-sarcoma-associated FTD (FTLD-FUS), a subtype of the behavioural variant of FTD.

**Conclusions:**

Our case highlights that despite the lack of specific FUS biomarkers the combination of clinical features and neuroimaging biomarkers can guide choosing the most likely differential diagnosis in a complex neurological case. Imaging in particular allowed an accurate measure of the topography and severity of neurodegeneration and the exclusion of AD-related pathology.

## Background

Frontotemporal degeneration (FTD) is a heterogeneous group of pathologies. It is the second most frequent cause of dementia in the presenile age range (< 65 years), secondary only to Alzheimer’s disease (AD), and accounts for 5–15% of all dementia. Progressive worsening in behavior, personality, and/or language, associated with a comparatively well-preserved memory, characterizes the clinical presentation. Chorea, that classically occurs in Huntington’s disease, an inherited neurodegenerative disease characterized by striatal atrophy, is a rather uncommon sign of clinical presentation in neurodegenerative diseases, even though the striatum has been reported as severely affected in FTD [1]. Chorea has previously only rarely been described in cases with FTD, notably as a clinical feature of the basophilic inclusion body disease subtype (BIBD) of fused-in-sarcoma-associated FTD (FTLD-FUS) [1].

Most FTD cases involve TDP-43 or tau-positive inclusions, but around 5-10% are due to FUS accumulation and classified as atypical FTD. Three rare forms of FTD are subtypes of FTLD-FUS: atypical FTLD-U (aFTLD-U), BIBD and neuronal intermediate filament inclusion disease (NIFID). Clinically, aFTLD-U is characterized by prominent obsessiveness, repetitive behaviors and rituals, social withdrawal and lack of engagement, hyperorality and marked stimulus-bound behaviour. NIFID and BIBD share several clinical features including dysarthria, motor neuron signs, parkinsonism, and memory impairment [1, 2].

We report the case of a patient presenting with amnestic mild cognitive impairment, chorea and severe atrophy and reduced metabolism of the left hemisphere on imaging, combined with mild [^18^F]flortaucipir uptake in the left caudate nucleus, leading to a diagnosis of probable FTLD-FUS pathology. Our case highlights that despite the lack of specific FUS biomarkers the combination of clinical features and neuroimaging biomarkers can guide choosing the most likely differential diagnosis in a complex neurological case.

## Case presentation

We report the case of a 75-year-old woman who was evaluated at the memory clinic of Geneva University Hospitals in 2023, with progressive memory loss for one year.

Medical history included former thyroidectomy with subsequent hypothyroidism, adequately managed with substitution therapy. Apart from the uninvestigated late onset cognitive impairment of the patient’s mother, family history was unremarkable for neurodegenerative diseases.

Neuropsychological exams showed multi-domain amnestic mild cognitive impairment (aMCI), mainly with episodic memory deficit, associated with minor deficiencies in executive functions and attention. The Mini-Mental State Examination (MMSE) was evaluated at 29/30, and the Montreal Cognitive Assessment (MOCA) at 23/30. The physical neurological exam revealed markedly asymmetric involuntary hyperkinetic movements affecting the distal right arm and the right leg, consistent with chorea. Occasional choreiform movements were observed in the left side and trunk. The patient herself did not complain about these involuntary movements. The rest of the exam was normal, with no evidence of parkinsonian features such as rigidity or bradykinesia, and no pyramidal or cerebellar signs were found. Language testing and behavioral assessment showed no significant abnormalities.

Cerebrospinal fluid (CSF) analysis was then performed and revealed a mild elevation of total tau and phospho-tau, as well as a normal Ab42 level, with total tau 521 ng/l (normal value, < 400 ng/l), p-tau 69.4 ng/l (normal value, < 56.5 ng/l), Ab42 2111 ng/l (normal value, > 725 ng/l), and Ab42/40 ratio 0.108 (normal value, > 0.069).

In addition, the plasma concentration of progranulin was also tested, and it was within the normal range with a value of 109 ng/mL, (normal value > 85 ng/mL).

A brain magnetic resonance imaging (MRI) performed in 2023 demonstrated severe left-sided supra-tentorial atrophy, associated with Fazekas grade 1 leukoaraiosis, both stable since a previous MRI realized one year before. However, a head CT scan performed in 2005 for suspected rhinosinusitis showed less significantly less marked atrophy, as compared to both MRI studies, speaking in favor of a progressive neurodegenerative disease. The MRI exams showed the asymmetrical atrophy to be particularly severe in the region of the left caudate nucleus, and evidenced important mineral deposits of the basal nuclei in the SWI sequence.

Two additional PET investigations were performed in 2023.

**Fig. 1:**
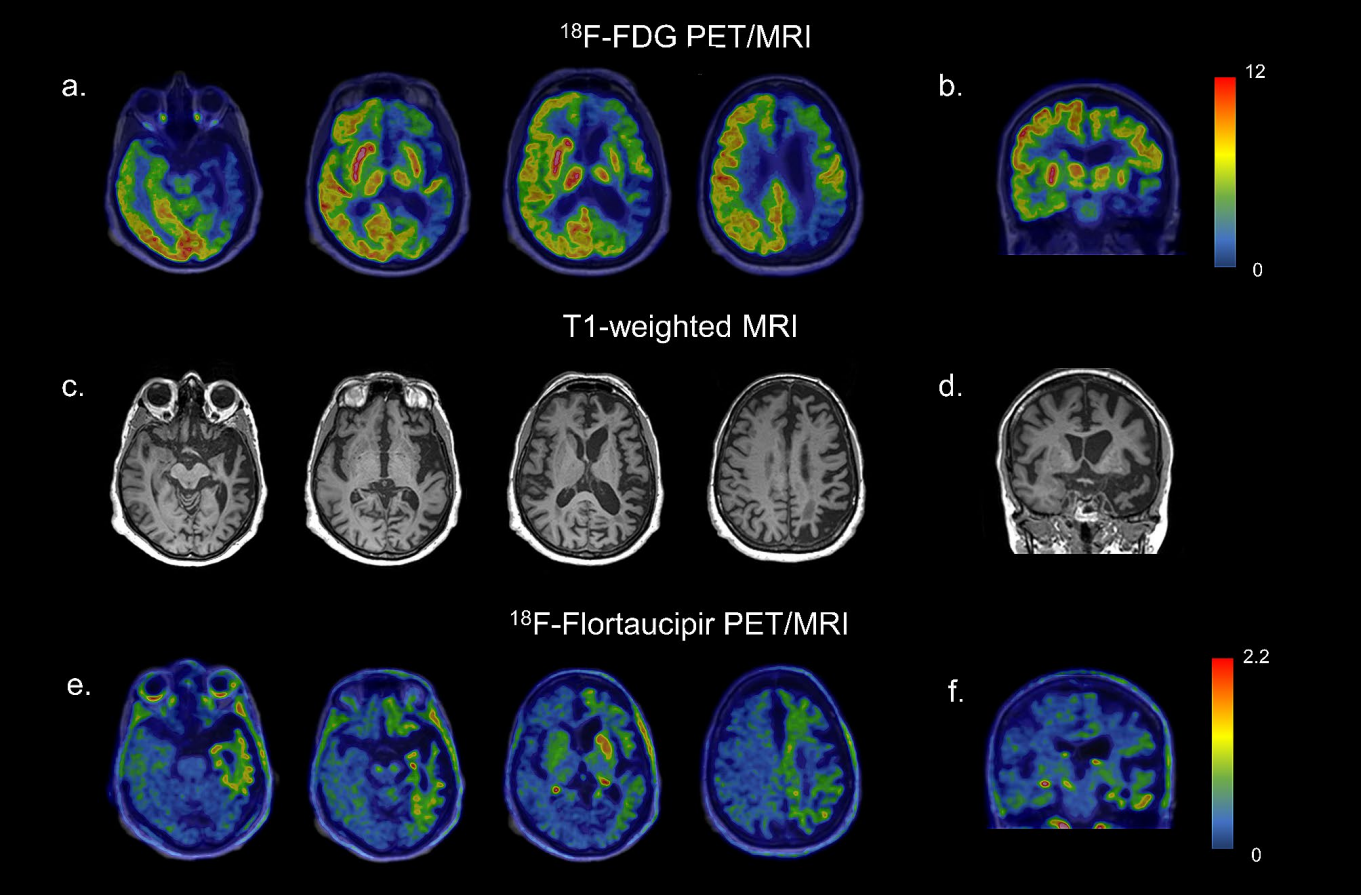
Axial **(a)** and coronal **(b)** slices of [^18^F]FDG PET showing severe asymmetric hypometabolism in the left frontotemporoparietal cortex and in the left caudate nucleus and in the left putamen. Axial **(c)** and coronal **(d)** T1-weighted MRI images show supra-tentorial asymmetric brain atrophy, predominating on the left side. Axial **(e)** and coronal **(f)** [^18^F]flortaucipir PET images show a mildly increased tracer uptake in areas characterized by hypometabolism.Images are in radiological orientation

[^18^F]FDG PET of the brain showed severe asymmetric hypometabolism in the left frontal and temporoparietal cortex, the left caudate nucleus, and, to a lesser extent, in the left putamen (Fig. 1a, b).

The patient underwent also a [^18^F]Flortaucipir tau PET study in the context of a research project of the memory clinic of Geneva University Hospitals. The visual assessment of the tau PET scan revealed an increased tracer uptake in areas characterized by hypometabolism as described before. Specifically, a weak neocortical uptake was found in the left frontal, parietal, temporal, and insular cortices, associated with a more intense uptake in the left caudate nucleus (Fig. 1e, f). According to current U.S. Food and Drug Administration statement, the increased tracer uptake in those areas was considered nonsignificant for AD, since neocortical uptake in the left frontal, parietal, temporal, and insular cortices did not reach a signal of more than 65% above the cerebellar mean. In the context of diffuse off-target binding, we found an asymmetric and moderate uptake in the left caudate nucleus, exceeding 65% of the cerebellar mean. No abnormalities were observed in the right hemisphere (Fig. 1e, f).

At the beginning, the diagnosis was opened between AD, FTD and corticobasal degeneration (CBD). Then, the imaging and CSF findings permitted to rule out an AD diagnosis and limited the differential diagnosis between CBD and a subtype of the FTD disease spectrum. CBD is a neurodegenerative tauopathy and one of the most common underlying pathologies of corticobasal syndrome (CBS), characterized by asymmetric rigidity, apraxia, dystonia, myoclonus, cortical sensory loss, and dystonia, as well as behavioral and cognitive impairments such as aphasia. FTD is a heterogeneous group of pathologies. It is the second most frequent cause of dementia in the presenile age range (< 65 years), secondary only to AD, and accounts for 5–15% of all dementia. Progressive worsening in behavior, personality, and/or language, associated with a comparatively well-preserved memory, characterizes the clinical presentation. FTD typically is divided into three clinical syndromes based on the predominant symptoms: the behavioral variant of FTD (bvFTD), and two types of primary progressive aphasia (PPA), namely a nonfluent variant (nfvPPA) or a semantic variant of PPA (svPPA). However, as the disease progresses, these phenotypes frequently converge [3].

MRI findings include in differential diagnosis also cerebral hemiatrophy syndrome, an uncommon disorder characterized in adults by unilateral brain volume loss, hemiparkinsonism, hemidystonia, hemiatrophy and, in exceptions, hemichorea [4]. These clinical features are not present in the presented case with the exception of chorea, asymmetrical but still bilateral.

Taking together the clinical picture and imaging exams allowed the hypothetical diagnosis of a neurodegenerative condition, namely a subtype of the bvFTD, called FTLD-FUS with some features of CBS. On the one hand, the clinical evaluation revealed markedly asymmetric choreiform involuntary movements and, on the other hand, neuroimaging results showed the severe left hemisphere atrophy, the left frontotemporoparietal cortex and the left caudate nucleus hypometabolism on [^18^F]FDG PET and the non-AD [^18^F]Flortaucipir tau PET pattern with an asymmetric and moderate uptake in the left caudate nucleus.

The presence of markedly asymmetric choreiform involuntary movements was an important feature in guiding the diagnosis. Chorea is usually characterized by excessive, spontaneous movements that are irregularly timed, nonrepetitive, randomly distributed, and abrupt. The classical form of chorea occurs in Huntington’s disease, an inherited neurodegenerative disease characterized by striatal atrophy. Chorea is a rather uncommon sign of clinical presentation in neurodegenerative diseases, even though the striatum has been reported as severely affected in FTD [1].

Chorea has previously only rarely been described in cases with bvFTD, notably as a clinical feature of the BIBD subtype of FTLD-FUS [1].

FUS is an RNA-binding protein with regulatory functions in the nucleus that accumulates pathologically in the cytoplasm in 5–10% of frontotemporal lobar degeneration (FTLD) cases, whereas TDP-43 or tau-positive inclusions are associated with the majority of FTLD cases.

Few neuroimaging studies assessed the in vivo molecular pathology and neurodegeneration in these cases due to the rarity of the pathology-proven cases during lifetime. Soleimani-Meigooni and coworkers [5] previously described tau uptake assessed by PET in a patient with a pathological diagnosis of FTLD-FUS, revealing intense uptake in the left caudate. Our reported case resembles this aforementioned tracer uptake, and also minimal tracer retention corresponding to areas of significant atrophy (Fig. 1e, f). The hypometabolism pattern described in our case adds molecular information to previous works about this rare entity.

[^18^F]Flortaucipir, formerly known as [^18^F]-T807 and [^18^F]-AV-1451, is one of the most widely used tau PET ligands. There is an excellent correlation between [^18^F]Flortaucipir retention and the distribution of neurofibrillary tangles in AD, but there have been inconsistent reports on its utility for non-AD pathologies.

In particular circumstances, the substrate of low-intensity tracer binding in these conditions is unknown. [^18^F]Flortaucipir may bind to TDP-43 or FUS aggregates at low levels in vivo, however, this does not persist in vitro after tissue preparation for autoradiography. The tracer might also detect other local pathological alterations, such as monoamine oxidase B expressed in reactive astrocytes or iron (or related proteins) in degenerating axons [5], resembling our case cortical uptake.

Augmented value can be conferred to our case through the integration of second-generation tau-PET tracers, which exhibit a superior off-target binding profile [6].

[^18^F]FDG PET results in FTLD-FUS cases have not been systematically described, however, the few described patterns of hypometabolism appear to correspond to the FTLD spectrum [7]. Goodwill and coworkers described a case of FTLD-FUS with corticobasal clinical features showing normal symmetric cortical FDG uptake but a markedly decreased FDG uptake in the caudate nucleus bilaterally [2]. In our patient, severe hypometabolism was found in the left caudate nucleus, associated with a significant uptake reduction in the gray matter of the left hemisphere. In accordance with a study from Joseph et al. [8], caudate atrophy on MRI appears to be significantly greater in FTLD-FUS compared with FTLD-TDP and FTLD-TAU, and the group suggests considering a diagnosis of FTLD-FUS in any patient presenting at a relatively young age, with behavioral variant frontotemporal dementia syndrome, negative family history, and severe caudate atrophy on head MRI, already in early stages of the disease course.

## Conclusion

To conclude, the neuroimaging results and the presence of chorea lead to a diagnosis of probable FTLD-FUS in the here presented case. The absence of other typical clinical features of FTLD-FUS, at the moment of presentation, could be explained by the fact that the patient was still in a prodromic phase (aMCI), and specific behavioral changes might also have been missed during neuropsychological evaluation. The impossibility of achieving a complete match between phenotype and molecular pathology is not surprising given the well-known fact that the same neuropathology can cause multiple clinical phenotypes or, conversely, different neuropathologies can cause comparable phenotypes. Moreover, the individual phenotype is determined by the interaction of pathology with multiple genetic and acquired factors, making the picture even more complicated.

Our case report highlights the importance of an integrated analysis of clinical features and neuroimaging results, including both the presence and topography of abnormalities, to reach a differential diagnosis also in the non-AD spectrum in the absence of specific molecular markers.

## Data Availability

Data sharing is not applicable to this article as no datasets were generated or analysed during the current study.

## Abbreviations

aMCI: amnestic Mild Cognitive Impairment
FTD: Frontotemporal Degeneration
CBD: Corticobasal Degeneration
CBS: Corticobasal Syndrome
FTLD: Frontotemporal Lobar Degeneration
BIBD: Basophilic Inclusion Body Disease subtype
MMSE: Mini-Mental State Examination
MOCA: Montreal Cognitive Assessment
CSF: Cerebrospinal Fluid
AD: Alzheimer’s Disease
bvFTD: behavioral variant of FTD
PPA: Primary Progressive Aphasia
nfvPPA: nonfluent variant of PPA
svPPA: a semantic variant of PPA
FTLD-FUS: Fused-in-Sarcoma-associated FTLD
MRI: Magnetic Resonance Imaging
aFTLD-U: atypical FTLD-U
NIFID: Neuronal Intermediate Filament Inclusion Disease
